# Supplementary material for: A systematic review to explore how exercise-based physiotherapy via telemedicine can promote health related benefits for people with cystic fibrosis
Source: PLOS Digit Health. 2023 Feb 27;2(2):e0000201. doi: 10.1371/journal.pdig.0000201 (PMC9970050; doi:10.1371/journal.pdig.0000201)
Supplement: Attachment — Submitted filename: Response to Reviewers.docx [file pdig.0000201.s002.docx]

**PDIG-D-22-00220 – A systematic review to explore how exercise-based physiotherapy via telemedicine can promote health related benefits for people with cystic fibrosis**

***Reviewer #1:***

**Below I provide section by section, critique of the shortcomings of the manuscript:**

***Response:*** We thank the reviewer for their time and effort in assessing the manuscript, and their comments below, which we address point-by-point.

**Eligibility Criteria: Some justification is needed for how and why the eligibility criteria was designed to be how it is.**

***Response:*** The specificity of the research question limited the eligibility criteria to an extent (e.g. only people with CF were examined, as this is a CF-oriented review). However, we are able to provide further reference to key clinical guidelines from the National Institute for Health and Care Excellence (NICE) which recommends that clinicians should routinely collate the outcomes we have selected (ppFEV1, QoL).

**Justification is needed for why only three databases searched and why were these three databases selected. specifically, PUBMED seems to be concerningly absent from the list of databases**

***Response:*** The selected databases were searched following consultation and guidance from research advisors at Birmingham City University, where the lead author (BB) initially undertook the work. Moreover, an initial scoping search of PUBMED did not highlight significant articles for inclusion and was therefore not systematically searched. This is however, discussed in the limitations.

**Selection Process: "A second reviewer (JML) performed the outcome of the searches" -- meaning of this sentence is unclear.**

***Response:*** Apologies for ambiguity. This meant “corroborated” and has been clarified within the manuscript.

**Selection Process: Authors need to address the fact that the initial search and screening was only conducted by one person -- this is highly unusual for systematic reviews. this needs to be addressed as a limitation of this study as this is major source of bias in this review.**

***Response:*** The initial search and screening were only conducted by one person (BB), as it was a solo MSc Physiotherapy dissertation submission in which authors were not entitled to have additional screeners. We have now acknowledged this is a limitation of the study and potential source of bias; however, this could not be avoided due to the circumstances. We do also make reference to the work of Waffenschmidt *et al.* (2019), who characterises the proportion of ‘missed’ studies in single v double screening, showing a median value as low as 5% - a value we believe is acceptable for this novel and emerging field.

Waffenschmidt *et al*., (2019). Single screening versus conventional double screening for study selection in systematic reviews: a methodological systematic review. *BMC Medical Research Methodology*, 19: 132.

**Study Risk of Bias Assessment: A risk bias assessment of the review itself needs to be conducted and reported. Consider ROBIS: https://www.ncbi.nlm.nih.gov/pmc/articles/PMC4687950/**

***Response:*** We acknowledge the reviewers suggestion, although as ROBIS is a intended as a review of systematic reviews, and not original primary studies. Therefore, we have retained the use of CASP within the present review.

**Effect Measures: Why were only ppFEV and CFQ-R data selected from the study? Simply the fact that all studies reported these is a shallow justification and does not give the reader a robust understanding of this decision.**

***Response:*** In alignment with our prior reference to clinical guidelines from NICE, the use of ppFEV1 and QoL are significant predictors of disease progression, and therefore this is why these were included (and subsequently why most studies will include these outcomes). We have detailed this within the ‘Effect Measures’ paragraph.

**3.3. Risk of Bias in Studies: The authors should provide more context for what the CASP score means; a mean score of 4.6 for single cohort studies does not mean much to a reader not familiar with CASP.**

***Response:*** We have provided additional information on the ‘Study Risk of Bias Assessment’ paragraph, detailing why CASP was chosen, and what maximal scores are available, in order to contextualise the results for the reader. We have also moved the CASP scores from the Supplementary File, to the main manuscript, in order to allow readers to contextualise the scores.

**3.4. Synthesis of Results: Authors should consider using sub-sections to structure their results synthesis section and thus adding more hierarchy to their reported results and framing the findings of the review more clearly.**

***Response:*** In line with the reviewers suggestion, we have included additional sub-headings, to include sections on ‘Study Characteristics’, ‘Intervention Characteristics’ and ‘Outcomes’.

**Authors should recognize that results of a review are not simply the outcomes of the reviewed studies but also their intervention and experimental designs. For example, In the results section, the authors should also report on how user adherence to telemedicine programs were measured and how this impacted the outcomes of the reviewed studies.**

***Response:*** We have included details on the interventions and experimental designs, as the reviewers suggests, and have further directed readers to Table 2, where this information is presented. We have also clarified the reported withdrawal from studies (*n* = 2 – 10).

**4. Discussion: The authors should use sections to structure their discussion and frame their argument.**

***Response:*** We have included a series of sub-headings within the discussion to help frame this further.

**The discussion sections requires significant work to be publishable. Authors need to clearly report on the state of this topic, address specific shortcomings, comment on the impact of the shortcomings on the lackluster results of the reviewed studies and suggest robust guidelines for future work in this area. Currently, the discussion sections reads more like a results sections, than their current results section. For instance, there needs to be discussion on the contents of the various Tables provided in this section in context of their impact on the reviewed studies and how they can be used to inform improved future studies.**

***Response:*** We have undertaken extensive re-writing, and re-structuring, of the discussion, including the incorporation of the above suggested sub-headings. We have elaborated further, in particular (as suggested), upon the context and implementation of interventions. In particular, we have noted how the pandemic has ushered in wider acceptance of remote exercise training in CF (Tomlinson *et al*., 2022), and how individualised exercise programmes are essential for people with CF (Williams *et al*., 2022).

Tomlinson *et al*., (2022). The impact of COVID-19 upon the delivery of exercise services within cystic fibrosis clinics in the United Kingdom. *Clin Resp J,* 16: 335-340.

Williams *et al*., (2022). The Exeter Activity Unlimited statement on physical activity and exercise for cystic fibrosis: methodology and results of an international, multidisciplinary, evidence-driven expert consensus. *Chron Resp Dis*, 19.

***Reviewer #2:***

**Thank you for this interesting and important topic, your manuscript is organized and written very well, and the discussion is strong. The only concern is that your manuscript sounds like a literature review of 8 articles but as you mentioned that it was because of a lack of enough studies in the literature.**

***Response:*** We thank the reviewer for their time and effort in examining the manuscript, and are pleased to hear they believe this is an organised and well written piece. We do acknowledge that the limited number of articles does preclude a more in-depth critique of the data, particularly given the heterogeneity in designs and outcomes. This has precluded our ability to undertake meta-analysis, which would enhance the discussion. However, as this is not possible, we have added this as a limitation in the discussion.

***Reviewer #3:***

**The health-related benefits of exercise-based physiotherapy via telemedicine is an important question to the CF community at this time. Post-pandemic, in CF centres across the UK physiotherapists continue to spend time developing and carrying out online-exercise programmes for people with CF in order to meet the changing needs of the CF population. It is so important that we begin to discuss and assess the value of these programmes so that we can begin to define what a quality CF physiotherapy telemedicine exercise programme looks like to inform our future strategy. This paper will therefore have broad interest within the CF community. The report shows excellent methodological design which is described in enough detail to be reproduced in future reviews. The conclusions are appropriate for the report's findings. The report is well organised, easy to read and understand.**

***Response:*** We thank Reviewer #3 for their time and effort in reviewing the manuscript, and are pleased to hear they feel it will have broad interest within the CF community.
